# Layer-by-Layer Delivery of Multiple Antigens Using Trimethyl Chitosan Nanoparticles as a Malaria Vaccine Candidate

**DOI:** 10.3389/fimmu.2022.900080

**Published:** 2022-08-17

**Authors:** Yang Xu, Ziyou Zhou, Brad Brooks, Tammy Ferguson, Judy Obliosca, Jing Huang, Izumi Kaneko, Shiroh Iwanaga, Masao Yuda, Yukiko Tsuji, Huitang Zhang, Christina C. Luo, Xunqing Jiang, Xiang-Peng Kong, Moriya Tsuji, Christopher K. Tison

**Affiliations:** ^1^ Luna Labs USA, Biotech Group, Charlottesville, VA, United States; ^2^ HIV and Malaria Vaccine Program, Aaron Diamond AIDS Research Center, New York, NY, United States; ^3^ Division of Infectious Diseases, Department of Medicine, Columbia University Irving Medical Center, New York, NY, United States; ^4^ Department of Medical Zoology, Mie University Graduate School of Medicine, Mie, Japan; ^5^ Research Institute for Microbial Diseases, Osaka University, Osaka, Japan; ^6^ Department of Biochemistry and Molecular Pharmacology, New York University (NYU) Grossman School of Medicine, New York, NY, United States

**Keywords:** chitosan, layer-by-layer, releases, malaria vaccine, multiple antigens

## Abstract

Developing a safe and effective malaria vaccine is critical to reducing the spread and resurgence of this deadly disease, especially in children. In recent years, vaccine technology has seen expanded development of subunit protein, peptide, and nucleic acid vaccines. This is due to their inherent safety, the ability to tailor their immune response, simple storage requirements, easier production, and lower expense compared to using attenuated and inactivated organism-based approaches. However, these new vaccine technologies generally have low efficacy. Subunit vaccines, due to their weak immunogenicity, often necessitate advanced delivery vectors and/or the use of adjuvants. A new area of vaccine development involves design of synthetic micro- and nano-particles and adjuvants that can stimulate immune cells directly through their physical and chemical properties. Further, the unique and complex life cycle of the *Plasmodium* organism, with multiple stages and varying epitopes/antigens presented by the parasite, is another challenge for malaria vaccine development. Targeting multistage antigens simultaneously is therefore critical for an effective malaria vaccine. Here, we rationally design a layer-by-layer (LbL) antigen delivery platform (we called LbL NP) specifically engineered for malaria vaccines. A biocompatible modified chitosan nanoparticle (trimethyl chitosan, TMC) was synthesized and utilized for LbL loading and release of multiple malaria antigens from pre-erythrocytic and erythrocytic stages. LbL NP served as antigen/protein delivery vehicles and were demonstrated to induce the highest *Plasmodium falciparum* Circumsporozoite Protein (PfCSP) specific T-cell responses in mice studies as compared to multiple controls. From immunogenicity studies, it was concluded that two doses of intramuscular injection with a longer interval (4 weeks) than traditional malaria vaccine candidate dosing would be the vaccination potential for LbL NP vaccine candidates. Furthermore, in PfCSP/Py parasite challenge studies we demonstrated protective efficacy using LbL NP. These LbL NP provided a significant adjuvant effect since they may induce innate immune response that led to a potent adaptive immunity to mediate non-specific anti-malarial effect. Most importantly, the delivery of CSP full-length protein stimulated long-lasting protective immune responses even after the booster immunization 4 weeks later in mice.

## 1 Introduction

Malaria kills over 260,000 children under five years old in Africa every year. The first malaria vaccine RTS,S/AS01 (Mosquirix^®^), an advanced recombinant protein-based vaccine was approved in 2021 for children under 5 years old. However, compared with other vaccinations, RTS, S/AS01 has only modest efficacy preventing approximately 30% of severe malaria cases after a series of four injections (1). The recombinant protein is a pre-erythrocytic stage circumsporozoite protein (CSP). It targets parasites before they can infect the liver, but this is only relevant for one stage of the parasite’s complex life cycle. One of the primary difficulties in malaria vaccination is the complexity of the multistage life cycle of *Plasmodium* and the intricate host-parasite interactions during the course of malaria infection. An optimal malaria vaccine would efficiently target multiple stages of the parasite life cycle (2). Further, vaccines offer another tool that could take pressure off continued use of combined malaria treatment drugs if one drug becomes resistant (2).

Due to the weak immunogenicity of investigational subunit vaccines, they often require advanced delivery vectors and/or the use of adjuvants (3). Design of novel adjuvant or nanoparticle delivery vectors that can stimulate immune cells and enhance vaccine efficacy has brought hope for future vaccine development. Improving the efficiency of vaccines by combination of adjuvants and advanced delivery systems based on controlled release technology is also one of the major priorities of the World Health Organization program for vaccine development (4). The goal is to develop a controlled release system to induce protective immune responses as soon as possible after the first immunization, while also providing prolonged immunity with negated or reduced administration of boosts.

To develop a more effective malaria vaccine with protective immune response and delivery of multiple life cycle stage antigens, we describe here a trimethyl chitosan-based layer-by-layer (LbL) nano-assembly vaccine platform (LbL NP) that enables the LBL delivery and release of multiple malaria antigens in a controllable manner. We have successfully constructed the LbL NP with efficient loading of different stages of antigens. It encapsulates a *Plasmodium falciparum* malaria parasite blood stage apical membrane antigen PfAMA-1 or merozoite surface antigen PfMSP-1 inside the core. The pre-erythrocytic stage antigen PfCSP (full length) is absorbed and stabilized on the shell layer of LbL construct. The size of the LbL NP vaccine candidates can be tuned from 200 nm to 400 nm, which are suitable for intramuscular injection (5). The highly positive charged surface of the trimethyl chitosan nanoparticles is beneficial for loading multiple antigens and confers greater solubility due to the trimethylation on the chitosan surface. A set of LbL NP was synthesized for encapsulation and loading with pre-erythrocytic and erythrocytic stage antigens at high efficiency of 70%-98%. The release of antigens was controlled between several days to months by tuning the charge and layer composition of the construct. The released antigens were characterized to verify that they maintained their stability and antigenicity. Most importantly, LbL NP served as antigen/protein delivery vehicles and induced the highest *Plasmodium falciparum* Circumsporozoite Protein (PfCSP) specific T-cell responses in mice, as compared to other adjuvants. Two doses of intramuscular injection with a longer interval (4 weeks) than other current vaccine candidates between them induced the high titer of humoral response against PfCSP. Furthermore, 5 of 6 mice were protected against a malaria challenge after receiving a booster of LbL NP delivery of full length of CSP as the vaccine candidate. Finally, general biosafety and dose tolerance studies demonstrated that LbL NP could be applied safely at less than 5 mg/kg with no significant adverse effects.

## 2 Materials and Methods

### 2.1 Materials

Chitosan (75–85% deacetylated, mol wt. 50–190kDa), Sodium tripolyphosphate (TPP), Tween 80 (Cat.8221870500, Sigma-Aldrich); Poly sodium 4-styrenesulfornate (PSS, average Mw~70k, Cat. 243051, Sigma-Aldrich), Sodium Hyaluronate (HA, average Mw~60k, Cat. 9067-32-7, Glentham)

### 2.2 Ethics Statement

All animal experiments were carried out in strict accordance with the Policy on Humane Careand Use of Laboratory Animals of the United States Public Health Service. The protocol wasapproved by the Institutional Animal Care and Use Committee (IACUC) at The Columbia University (Animal Welfare Assurance no. D16-00003) and Michigan State University (Animal Welfare Assurance no. A3955-01). In addition, all components of the University are accredited by the Association for Assessment and Accreditation of Laboratory Animal Care, International (AAALAC Unit #1047).

### 2.3 Animals

In the immunogenicity and efficacy studies, female BALB/c mice 8-10 weeks of age were purchased from The Jackson Laboratory (Bar Harbor, ME) and maintained under specific pathogen-free conditions. We only used female BALB/c to determine the infectivity of the transgenic PfCSP/Py parasites, as shown in previous published literature as a reference (6). In the safety studies, Sprague-Dawley rats were obtained from Charles River Laboratories and weighed 234-272 g at the time of use. Animals were individually housed, with free access to standard rodent chow and fresh water throughout the study. Animals were maintained on an automated 12/12-hour light/dark cycle with 7:00 am as the start of the light phase.

### 2.4 Parasites

We have previously generated the transgenic parasite, PfCSP/Py Spz by inserting a construct expressing the PfCSP at the locus of the *P. yoelii* CSP gene by double cross-over homologous recombination (7). The PfCSP/Py parasite, which is a useful tool to evaluate human malaria vaccine based on PfCSP in a mouse model was then shipped to Sanaria Inc., where the parasites were purified and cryopreserved. We focused on PfCSP/Py Spz challenge study to demonstrate the LbL NP delivery efficacy in this paper.

### 2.5 Expression and Purification of the Recombinant PfCSP, PfAMA-1 and PfMSP-1

The recombinant PfCSP was expressed in bacteria as reported by Zhang et al. (7). Briefly, the PfCSP plasmid (synthesized by Genscript) was transformed into the BL21(DE3) E. coli strain. The construct was subcloned into the *E. coli* pET-11a expression vector downstream of the T7 promoter using the NdeI and Bam HI restriction sites. The resulting transcribed gene incorporates additional amino acid sequence of HHHHHHHH at its 3’ end and the PfCSP expression was induced using isopropyl 1-thio-β-galactopyranoside (IPTG, 1mM) at 20°C when the culture reached an optical density of 0.5 at 600 nm. Cells from the overnight culture were pelleted by centrifugation, resuspended in lysis buffer, and passed through a French press three times. The lysate was centrifuged for 30 min at 10,000 × *g* to pellet down the inclusion bodies and cellular debris. The PfCSP was purified using Ni-nitrilotriacetic acid (Ni-NTA) affinity chromatography. Nickel-captured CSP was then refolded in a refolding buffer and was subsequently dialyzed and concentrated. The gel image was captured in order to check the MW of produced CSP. The final yield was around 5 mg from a liter of LB culture with purity greater than 95%.

The recombinant AMA-1 and MSP-1 proteins were produced by mammalian cells. Briefly, the AMA-1 and MSP-1 genes were codon optimized for mammalian cell expression and synthesized by a commercial vendor (Genscript, NJ) with a secretion signal and a C-terminal 8x His tag. They were then cloned into the modified expression vector pVRC8400 (kindly provided by the Vaccine Research Center, National Institutes of Health). Plasmid DNAs encoding these proteins were transiently transfected into Freestyle 293-F cells, cultured in Erlenmeyer flasks using 25 to 30% of the nominal volume, and rotated at 120 rpm under standard humidified conditions (37°C and 5% CO2). Cells were allowed to secrete the malaria proteins for 4-5 days. Cell supernatants were filtered and loaded onto Ni-NTA beads, and proteins were eluted with 250 mM imidazole. Proteins were buffer exchanged into PBS by dialysis.

### 2.6 Statistical Analyses

Data was collected on an Excel spreadsheet. Graphic interpretation of the data was performed using GraphPad Prism 9.0.0. Statistical comparisons of vaccinated groups to vehicle control were made using Kruskal-Wallis one-way analysis of variance followed by post-hoc testing using Dunn’s multiple comparisons test. In all cases, a p<0.05 was considered statistically significant.

### 2.7 TMC Synthesis and Characterization

Trimethyl chitosan (TMC) samples with different degrees of quaternization were synthesized according to a previous method (8), with some modifications of the reaction time. Briefly, chitosan was methylated by methyl iodide in a strong base (NaOH) solution at 60°C for a single time or multiple times to obtain TMC with different degrees of quaternization. The products were dissolved in NaCl solution and then purified by dialysis against the water and then lyophilized to be ready for next step encapsulation of antigens. The purified products were then analyzed by 1H NMR spectroscopy. The FTIR spectra of TMC and chitosan were measured using Nexus 6700 FTIR with Diamond ATR insert.

### 2.8 TMC-TPP Nanoparticle and TMC-TPP-Protein Nanoparticle Synthesis, Loading, and Release Tests

The TMC nanoparticles were prepared by ionic gelation of TMC with TPP anions. 10 mg of TMC was dissolved in 5 ml of water to make a 2 mg/ml solution. Subsequently, 2 ml of TPP solution at concentrations (2 mg/ml) was added drop-by-drop to the above solution under magnetic stirring at room temperature for 0-60 min. To optimize the synthesis parameters of LbL NP, we first chose BSA as a model protein for the load and release test since it has similar size of targeted malaria proteins. And then, we used targeted CSP, AMA-1 and MSP-1 proteins for the test. Protein was first dissolved in the TMC solution for stirring of 15 min before adding the TPP. The size and zeta potential of the TMC nanoparticles were measured with a Zetasizer (Nano ZS90, Malvern Panalytical). The particle-size distribution of the nanoparticles is reported as a polydispersity index (PDI). All measurements were performed in triplicate. The loading efficiency and capacity of the protein loaded TMC nanoparticles were determined by separating the nanoparticles from free proteins by centrifugation at 18,000×g for 15 min. The amount of free/unloaded protein in the supernatant was measured by micro-BCA protein assay. The supernatant of non-loaded TMC nanoparticle suspension was used as a blank. The loading efficiency and loading capacity of the nanoparticles were calculated as follows and all measurements were performed in triplicate.


LE(%)=(total amount of protein−free protein)/total amount of protein×100%

LC(%)=(total amount of protein−free protein)/nanoparticles dry×100%

To identify and quantify loading and release of different proteins in LbL NP, we used fluorescence dye labelled technology for protein quantification. 2 mL of TMC solution was mixed with 1 mL and 0.5 mL of Texas-red 594-protein solution (Thermo Fisher, 1 mg/mL) in separated glass vials. Into each mixture solution, 50 µL of Tween 80 was added as non-ionic surfactant. After stirring for 10 minutes to fully mix TMC, BSA (or CSP, or AMA or MSP), and Tween 80, 2 mL of TPP solution (2 mg/mL) was slowly added under constant stirring. After reaction for 1 hour, the reaction solutions were purified by gradient centrifugation with 10 µL of glycerol three times. The samples were then redispersed into DI water, and the second layer of protein labelled by Alexa Fluor 488 (Thermo Fisher) was added with or without a protection layer of polystyrene sulfonate (PSS) or Hyaluronate (HA). The purified NPs were redispersed in PBS immediately for burst release testing at 37°C and the supernatant was also collected from the purification to calculate the encapsulation efficiency/burst release. NPs were kept in PBS at 37°C in an orbital shaker for up to 5 weeks to monitor the releases. During each period of data collection, old PBS buffer was exchanged with new buffer and the released protein was measured by fluorescence and UV-Vis spectrometry. Dynamic Light Scattering DLS and Zeta potential (Zetasizer Nano ZS90, Malvern Panalytical) were used for NP size distribution and surface charge at each step of formation of LbL NP or LbL NP-protein complexes.

Vaccine candidate preparation: After determining the composition of LbL NP using BSA as the model protein, the malaria protein PfCSP, PfAMA-1, and PfMSP-1 were encapsulated or absorbed on the LbL NP using these methods. Trimethyl chitosan (TMC) was dissolved in DI water at a concentration of 1 mg/mL, and tripolyphosphate (TPP) was dissolved in DI water at a concentration of 1 mg/mL. 1 mL of TMC solution was mixed with 250 µL of PfCSP solution at a concentration of 1 mg/mL. After TMC/PfCSP interaction for 30 minutes, 200 µL of TPP solution was added into the TMC/PfCSP mixture to form LbL NP CSP. After reacting for 1 hour, the reaction was stopped from stirring, and the solution was purified by centrifugal filtration. The final product was fully purified and then redispersed into 500 µL of sterile PBS.

For the three protein LbL formulations, TMC was dissolved in DI water at a concentration of 1 mg/mL, and tripolyphosphate (TPP) was dissolved in DI water at a concentration of 1 mg/mL. 1 mL of TMC solution was mixed with 91 µL of PfAMA-1 solution at a concentration of 1.64 mg/mL and 181 µL of PfMSP-1 solution at a concentration of 0.83 mg/mL. After mixing TMC and PfAMA-1/PfMSP for 30 minutes, 200 µL of TPP solution was added into the TMC/PfAMA-1/PfMSP mixture to form LbL NP AMA-1/MSP. After reacting for 1 hour, 150 µL of PfCSP solution at a concentration of 1 mg/mL was added to form LbL NP AMA-1/MSP-1/CSP formulation. After reacting for another 1 hour, the reaction was stopped, and the solution was purified by centrifugal filtration. The final product was redispersed into 300 µL of sterile PBS. The unloaded nanoparticles were synthesized using the same method which will be used as control. The final volume was concentrated to 500 µl.

Adjuvant Montanide formulation preparation: 250 µL of PfCSP solution at a concentration of 1 mg/mL was concentrated by using a protein concentrator (molecular weight cutoff 30K, Thermo Scientific™ Pierce™). Into the concentrated CSP solution (volume was ~20 µL), sterile PBS was added to achieve a final volume of 150 µL. Then, CSP solution was mixed with 350 µL of Montanide ISA 720 VG ST solution using two syringes for obtaining water in oil emulsion, for a final volume of 500 µL. A similar process was used for the three-malaria antigen formulations. 152 µL of AMA-1 at a concentration of 1.64 mg/ml, 250 µL of CSP at a concentration of 1 mg/ml, and 301 µL of MSP-1 at a concentration of 0.83 mg/ml were mixed and concentrated using the protein concentrator (Thermo Fisher, 10K, MWCO) by centrifugation. Into the concentrated protein solution (volume was ~20 µL), sterile PBS was added until the final volume was 150 µL. Then, 350 µL of ISA720 VG ST solution was emulsified with the protein solution. The final volume of solution was 500 µL and was transferred to an empty vial. As the control group, 150 µL of PBS was emulsified with adjuvant Montanide ISA 720 VG ST to obtain final volume of 500 µL.

Adjuvant 7DW8-5 formulation preparation: 250 µL of CSP solution at a concentration of 1 mg/mL was added of 225 µL of PBS which brought the total volume to 475 µL. Subsequently, 25 µL of 7DW8-5 solution at a concentration of 1 mg/mL was added into CSP solution. The final volume was 500 µL. Similar to CSP protein formulation, 152 µL of AMA-1 at a concentration of 1.64 mg/ml, 250 µL of CSP at a concentration of 1mg/ml, and 301 µL of MSP-1 at a concentration of 0.83 mg/ml were mixed and concentrated using a protein concentrator (molecular weight cutoff 30K). Into the concentrated protein solution (volume was ~20 µL), sterile PBS was added until the final volume was 475 µL. And then, 25 µL of 7DW8-5 solution at a concentration of 1 mg/mL was added into mixed three protein CSP/AMA-1/MSP-1solution. The final volume was 500 µL. As the control group, 475 µL of PBS was added with adjuvant 7DW8-5 to obtain final volume of 500 µL.

### 2.9 Antigenicity and Binding Kinetics Measurements of LbL NP CSP Complex Using nanoSPRi Platform

An antigenicity test was used for evaluation of released protein to identify its antigenicity. The method is an enzyme-linked immunosorbent assay (ELISA). Malaria antigens were prepared at concentrations of 20 µg/mL and 10 µg/mL in PBS, pH 7.4. 100 µL of each preparation were added in duplicate to wells of a 96-well ELISA plate and incubated at room temperature for 1 hour. Plates were washed in PBS-Tween 5 x. 300 µL of blocking buffer was added to each well and incubated overnight at 4C. Plates were washed in PBS-Tween 5 x. 100 µL of 10 µg/mL primary antibody in PBS was added to each well (excluding no-antibody control wells) and incubated at room temperature for one hour. Plates were washed in PBS-Tween 5 x. 100 µL of a 1:2000 dilution of goat anti-human HRP IgG secondary antibody was added to each well (excluding no-antibody control wells) and incubated at room temperature for 1 hour. Plates were washed in PBS-Tween 5 x. 100 µL of TMB substrate was added to each well and color allowed to develop for ~ 25 minutes. 100 µL of 2 M sulfuric acid was added to each well and readings taken at OD450 nm.

The construction of nanoSPRi platform for malaria protein evaluation involves a sandwich assembly of malaria’s antibody/antigen matched pair. In this design (details provided in Results and **Figure S1**), a capture antibody (CAb, e.g., anti-CSP) array was immobilized on the chip. The chip was prepared with 5 spots (repeats) for each sample (5-10 samples) to enable multiplex antigen detection. A sample containing malaria antigen (e.g., PfCSP) was then injected in the flow cell. The antigen was then specifically bound with the CAb array. The biotinylated detection antibody (e.g., biotinylated anti-CSP) was used as the detection system for bound antigen while streptavidin-coated nano enhancer quantum dots (QDs) were used as the signal amplification technique. When a QD is covalently bound to the detection antibody *via* biotin-streptavidin interaction, QD adds mass to the sandwich construct resulting in appreciable SPRi signal detection. The SPRi measurements were carried out as follows. First, the sensing chip was functionalized with thiolated protein A solution for 2 hrs in a humidity chamber (65-75% relative humidity). Thereafter, the chip was washed with deionized (DI) water, dried with a nitrogen stream, and allowed to form a self-assembly layer of protein A on the chip surface for at least 3 hrs prior to use. The functionalized chip was then be spotted with anti-malaria capture antibody (3C1 or 2A10) in 5 replicates for 4 different concentrations (125, 250, 500, and 1000 µg/mL). The resulting spots were then incubated inside a humidity chamber for at least two hours. The chip was then washed with DI water, air dried by gentle stream of nitrogen, mounted onto the SPRi instrument, and treated with Luna Labs’ proprietary blocking agents. The instrument was (16) calibrated using a high salt concentration (25 mM NaCl) before analysis. Analysis was done by first injecting 1× PBS buffer to obtain a baseline signal. A single injection of 10 ng/mL of released PfCSP protein solution or control PfCSP original soultion was introduced in the flow cell at a running buffer flow rate of 20 µL/min to allow effective capture of the antigen by immobilized antibody spots on the sensing chip surface. The obtained sensorgrams were globally fit to a 1:1 biomolecular interaction model (software: SrubberGen, HORIBA Scientific) to calculate binding kinetic parameters: k_a_, k_d_ and K_D_.

### 2.10 Safety Studies

We evaluated the tolerability of a trimethylated chitosan nanoparticles administered intramuscularly to male Sprague‐Dawley rats twice over 14 days. In total, sixteen male Sprague-Dawley rats were assigned to 4 groups (vehicle control or three dose levels of nanoparticle [n=4/group]) as shown in **Table S1**. Clinical observations were recorded up to once daily and body weights were assessed prior to dosing and at least twice weekly thereafter. The tissues/organs were also collected and weighed from all animals: heart, liver, kidney, and muscle tissues at the site of administration. Tissues/organs were processed using standard H&E staining. Microscopic evaluations of tissues/organs were conducted by a qualified veterinary pathologist.

### 2.11 Immunogenicity Studies

We formulated vaccine candidates by loading one pre-erythrocytic protective antigen PfCSP and two blood stage antigens PfAMA-1 and PfMSP-1 in the LbL NP structure as described in section 2.3. Three formulations were delivered for the animal studies for either two or three doses by intramuscular injections to compare the vaccine candidate performance (**Table 1**). They were LbL NP-CSP (TMC-TPP encapsulated PfCSP); LbL NP-AMA-1/CSP-1 (TMC-TPP encapsulated AMA-1 inside of core, and PfCSP in the outside layer); LbL NP-CSP/AMA-1/MSP-1 (TMC-TPP encapsulated MSP-1 with second layer of AMA-1 and the outside layer is CSP). Additionally, another two adjuvants Montanide ISA 720 [Seppic Inc. (9)], a natural metabolizable nonmineral oil and a highly refined emulsifier of mannite monooleate family, and 7DW8-5 (10), a recently identified novel analog of α-galactosylceramide (α-GalCer) that enhances the level of malaria-specific protective immune were used to compare and incorporated with these LbL NP formulations.

**Table 1:** Vaccine candidate formulation compositions in Immunogenicity Study. 1-12 group animals were injected by two doses of vaccine candidates, but 13-24 group animals were injected by three doses.

Groups:	Immunization regimens
1, 13	LbL NP-CSP + ISA720
2, 14	LbL NP-AMA-1/CSP + ISA720
3, 15	LbL NP-CSP/AMA-1/MSP-1 + ISA720
4, 16	LbL NP + ISA720 only
5, 17	LbL NP-CSP + 7DW8-5
6, 18	LbL NP-AMA-1/CSP + 7DW8-5
7, 19	LbL NP-CSP/AMA-1/MSP-1 + 7DW8-5
8, 20	LbL NP + 7DW8-5 only
9, 21	LbL NP-CSP
10, 22	LbL NP-AMA-1/CSP
11, 23	LbL NP-CSP/AMA-1/MSP-1
12, 24	LbL NP only

In total, 24 female BALB/c mice (n=4 per group) were immunized intramuscularly with each formulation twice or three times at a 3-week interval to determine the vaccine candidate potential dosing. For each animal, an injection containing 10 µg of protein was administered for each dose. Three weeks after the boost, mouse sera were collected for serology analysis of the antibody titers of PfCSP, PfMSP-1, and PfAMA-1 for each formulation and the numbers of IFN-γ-secreting T cells in spleens of mice immunized with antigens by intramuscular injection was measured by IFN-γ enzyme linked immunospot (ELISPOT) assay (10).

### 2.12 Efficacy Studies

We prepared nine vaccine candidate samples for nine group of animals with two doses for each group of animals and one group using unloaded LbL NP as a control. These samples included two control adjuvant groups (Montanide ISA 720 VG ST and 7DW8-5). 10 groups (n=6 for each group, nine vaccine candidates and one control) of 8-10 weeks old female BALB/c mice were immunized intramuscularly with each formulation twice with a 4-week interval between two doses. For each animal, an injection containing 10 µg of protein was applied for each dose. Four weeks after the two doses, naïve as well as immunized mice were challenged with 1000 transgenic PfCSP/Py sporozoites intravenously. The infectivity of PfCSP/Py Spz was determined by the presence or absence of parasites (parasitemia) in the blood of the challenged mice. This was done by way of microscopic examination of Giemsa-stained thin blood smears made from one drop of blood extracted from the tail vein of the mice from day 4 to day 12 post-Spz challenge.

## 3 Results

### 3.1 TMC Precursor Synthesis Optimization and Characterization

**Figure 1:**
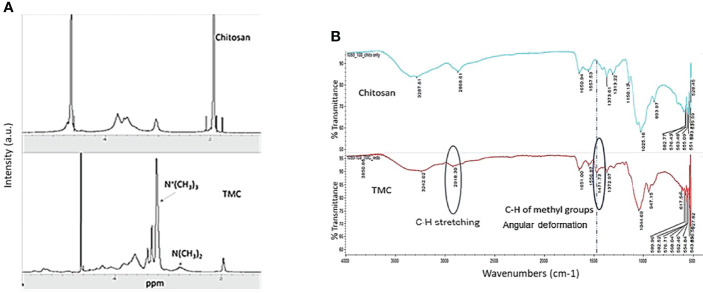
**(A)** Characteristic 1H-NMR spectrum of Chitosan and N-trimethyl chitosan (TMC) in the D2O. **(B)** FTIR spectra of Chitosan and N-trimethyl chitosan (TMC).


The critical component of the LBL vaccine delivery platform is trimethyl chitosan (TMC), which allows antigens to be properly loaded to form nanoparticles. We first synthesized TMC from chitosan which creates a positively charged molecule that is soluble in aqueous solutions. To accomplish this, we performed a methylation reaction two times to achieve tri-methylation. The proton nuclear magnetic resonance (1H-NMR) spectrum of TMC characterization was shown in **Figure 1A**. According to the literature (11), the signal at 3.22 ppm corresponds to the methyl group at the N, N, N-trimethylated site, the signal at 2.72 ppm corresponds to the methyl group at the N, N-demethylated site, and the signals ranging from 4.8 to 5.4 ppm were attributed to the hydrogen atom bonded to the carbon 1 of the glycoside ring. The degree of quaternization (DQ) was calculated as about 50.5% using the following equation:

$$\text{DQ}\left(\%\right)=\left[\left(\frac{\int \text{TM}}{\int \text{H}}\right)\times \frac{1}{9}\right]\times 100$$

where ʃ_TM_ was the integral of the trimethyl amino group (quaternary amino group) peak at 3.3 ppm and ʃ_H_ was the integral of the 1H peaks from 4.7 to 5.7 ppm. The DQ was used for evaluation of trimethylation degree every time after tri-methylation and also serves as the quality control factor for TMC production.

The FTIR band shown in the **Figure 1B** at 1,471 cm^−1^ was attributed to angular deformation of C-H bonds of methyl groups existing in higher proportion in TMC (8), as compared with chitosan only. The bands at 2,918 cm^−1^ that appear in the FTIR spectrum of TMC were attributed to characteristic stretching of C-H bonds. The FTIR spectrum further demonstrated that we successfully performed the trimethylation of chitosan.

### 3.2 Parameter Determination and Optimization for LbL NP-Antigen Formulation, Antigen Loading and Release

**Figure 2:**
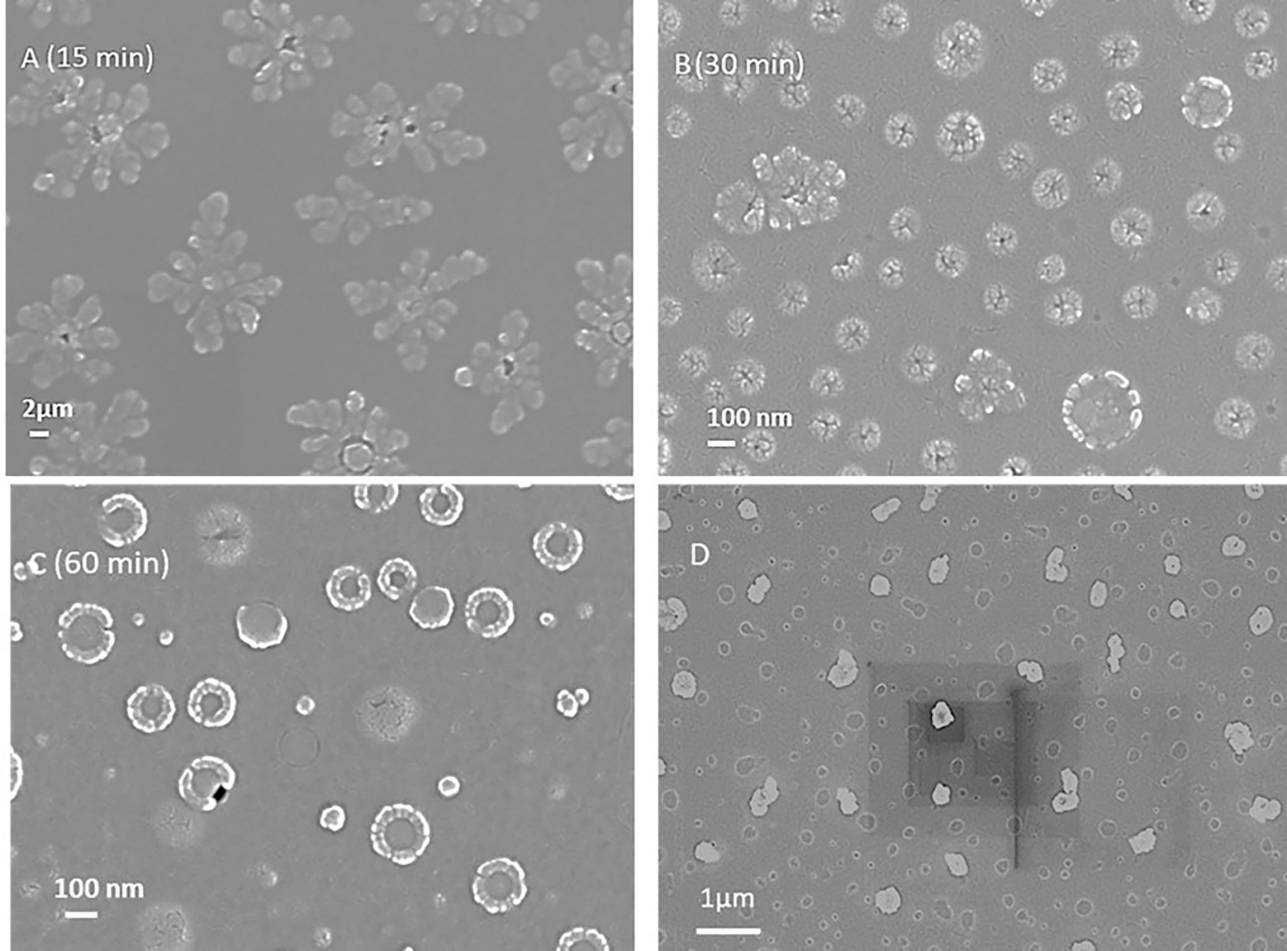
**(A-C)** Scanning electron microscope images of LbL NP formations at different reaction time (15, 30 and 60 min). 60 min was found to be the optimized time for the core-shell structural nanoparticle formation. **(D)** SEM image of LbL NP encapsulated with protein.

To form TMC LbL NPs, a cross linker is required. Tripolyphosphate (TPP) is a non-toxic polyanion that can crosslink with TMC to form uniform nanoparticles under certain condition. By using a cross linker, the encapsulation and loading of multiple antigens on the TMC nanoparticles can also be achieved. For making nanoparticles only, we mixed TMC and TPP solution as indicated in the Methods Section. 60 min of reaction time was selected for core-shell layer structural nanoparticle formation (**Figure 2**). After 15 min of reaction, the particles were starting to form. After 30 minutes of reaction time, the nanoparticles were formed at approximately 50 nm diameter as a single nanoparticle, though they also self-assembled to start forming the core-shell layer structure nanoparticles at a size range of 200-300 nm (**Figure S2**). After 30-60 minutes, the nanoparticles were found to be stable in the solution. Zeta potential analysis was used paralleled with DLS for measuring surface charge of nanoparticles during the synthesis. TMC precursors had a highly positive charge of +40~+50 mV, and TMC-TPP nanoparticle formation lowered the surface charge to approximately +20~+30 mV due to the TPP crosslinking.

In addition to the TPP-crosslinked TMC nanoparticle “core,” we also investigated the another negatively charged long chain polymer PSS as the crosslinker to compare with TPP. As shown in **Table S2**, the charge of NPs changed from negatively charged to positively charged when the ratio of TMC : PSS decreased from 0.91: 1 to 0.83: 1. As a result, we focused on those composition that has ratios higher than 1:0.83 for the protein loading since it requires a positively charged surface. At the same time, we investigated the charge changes when we included both TPP and PSS in the composition and tuned the ratio between TMC : TPP to PSS. As shown in **Table S3**, once we added PSS during NP formulation, the surface charge of the NP significantly decreased from highly positive charge + 23 mV to + 4 mV. Also, we observed the presence of white aggregates formed during the NP synthesis after adding higher amount of PSS (TMC : PSS ratio >10:1). These results indicated that adding the PSS could cause aggregates of NPs if the amount is high. As a result, we worked to optimize the amount of PSS used in the composition to form stable NPs without precipitation. Since PSS has shown stronger interaction with TMC. At the same time, another negatively charged polymer hyaluronate (HA, 60K MW), was selected for comparison with PSS as an alternative option for the outer-layer coating. Different amounts of HA were tested similarly to that performed for PSS. However, we did not see aggregates during the LbL NP formation which indicated that HA would be a good candidate for LbL NP formulation.

We next evaluated protein loading and encapsulation inside LbL NP. First, bovine serum albumin (BSA) was selected as the model protein for nanoparticle loading and release tests due to the size similarity to the malaria antigens targeted in this study to obtain preliminary data. We could encapsulate BSA in TMC-TPP nanoparticle with size of 320 ± 82 nm which is larger than the pure NPs (224 ± 20 nm) as indicated in **Figure S2**. Also, the empty core was encapsulated with protein which showed a fully solid nanoparticle in SEM image (**Figure 2D**).

**Figure 3:**
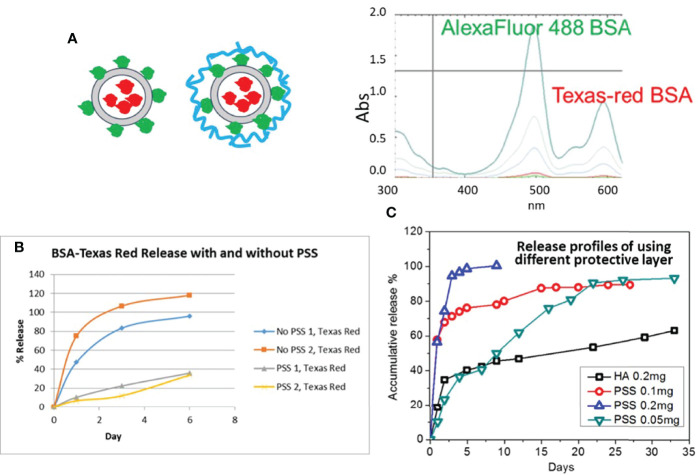
**(A)** Schematic of dye Alexa Fluor 488 (green) and Texas-red labelled BSA LbL loading on NPs (red circle) by two approaches without and with second protection layer coating (Blue); UV-Vis spectra of two dye labelled protein mixture. **(B)** Release profile of NP formulation with and without PSS as the protective layer. **(C)** Release profile of LbL NP formulation with different mass amount of PSS or HA as the protective layer.

We chose two different dyes for the labelling of BSAs to present different proteins in the LbL encapsulation and loading test. Once we determined the optimized composition of LbL NP using these model proteins, we worked on the malaria antigen proteins. One BSA was labelled with Texas Red dye (abs: 594 nm) for encapsulation in the core of the chitosan LbL NP, and a second BSA with Alexa Fluor dye (abs: 488 nm) dye labelling was immobilized on the NP surface without and with adding the outer protection layer (blue circle) polystyrene sulfonate (PSS) or hyaluronate (HA) as shown in the **Figure 3A**. The two dye-labelled BSA proteins can be identified separately using UV-Vis spectroscopy, as shown in **Figure 3A**. Results indicated that there was no interference between the emission peak of each dye when they were mixed in solution and measured simultaneously.

The loading efficiency (LE) of core protein encapsulation was calculated at approximately 85.2%. However, during the loading process for the second layer protein, the first layer of protein exhibited a burst release. The LE slightly decreased to 82.3% without a protection layer of PSS, while it further decreased to 74.2% with PSS coating. However, the loading efficiency of the second layer protein was approximately 91.2% without PSS and 84.5% with PSS. Also, from the release test we could see that the encapsulated protein was released significantly faster as expected, so the protective layer is necessary to obtain a long-term release profile (**Figure 3B**).

Secondly, to achieve the release profile for each protein layer, we optimized the composition of the second layer of PSS and TPP. We determined that the ratio of TMC : TPP:PSS should be kept below 1:0.2:0.5 for protein loading. The final formulation loading efficiency of protein can be reached as high as 91.2% or 97.5% when we applied TMC : TPP:PSS ratio of 1:0.2:0.2 or 1:0.2:0.1 respectively. Similar to PSS, HA used as the outer layer was also evaluated for effect on protein release. The test for the selected formulation release were monitored for more than a month, with results shown in **Figure 3C**. The burst release of the Texas Red BSA protein for these samples was 30-40% for using higher PSS contents (0.1 mg), but when we decreased the amount of PSS to 0.05 mg or for 0.2 mg of HA coating, the burst release decreased to 10% and 24%, respectively. Also, the encapsulated protein was released significantly faster with higher content of PSS layer, and the release rate decreased with a reduction in the amount of PSS used. For achieving a month interval release profile, we need to limit the coating amount between 0.05-0.1 mg per 1mg TMC. HA used as the coating for the outer layer provided even longer protein sustained release for if we need to achieve greater than one month release profile.

**Figure 4:**
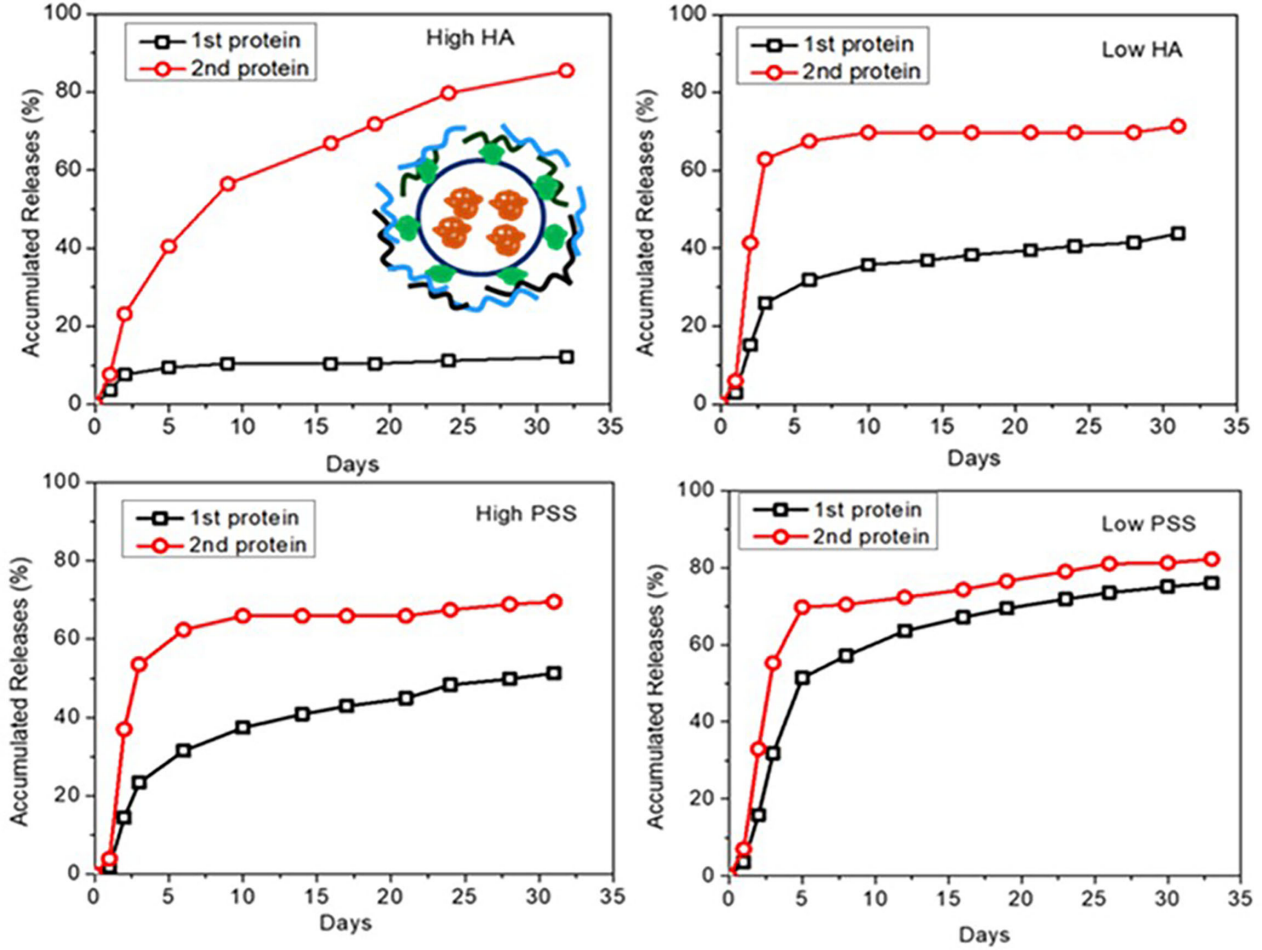
Dye-labelled protein release profiles for different formulations of LbL NP by tuning of amount of outside of PSS or HA protective layer (insert is the schematic for two layers of loading of dye labelled proteins by coated with protective layers).

At the final optimization, we chose the loading and release of a second layer of BSA using 0.05 mg (High) and 0.01 mg (Low) of PSS or HA as the protective layer. When we used PSS, the encapsulation efficiency for BSA was as high as 98.5% and 93.4%, for the inside (red) and outside (green) layers, respectively (**Table 2**). Results also show that over the first 4 days, both protein layers have similar release profiles (**Figure 4**). However, after a week of release, the outer layer of protein (“2^nd^ Protein”) demonstrated an increased release rate as compared to the inner core layer of protein (“1^st^ protein”). It was clear that when less protective polymer used (0.01 mg), there is a faster release rate. However, for HA coated LbL NP formulation, the inner layer (first protein) release reached 42% of total after 30 days for samples with lower amounts of HA protective layer, as compared to only 15% of release when we used higher amounts of protective coating of HA. However, the second protein was almost entirely released for higher HA samples. For the PSS protective coating samples, the release was higher than 80% at 30 days. However, the higher PSS coating, the first protein was released at a lower amount (40%) compared to low PSS coating with almost 80% released. These results indicated that we could control the protein release profile by tuning the protective layer of coating amount and compositions. Using BSA as the model protein, we have proven the concept using LbL NP for multiple antigen loading/encapsulation and delivery. We used this model to apply for malarial antigen candidates pre-erythrocytic protective antigen CSP and blood stage protective antigen AMA-1, MSP-1 with further investigation and animal studies. The loading efficiency for these three proteins were in the range of 70-98%.

**Table 2:** LbL NP composition and encapsulation and loading efficiency for each of the vaccine candidates.

Sample	TMC (mg)	TPP (mg)	PSS (mg)	HA (mg)	BSA (red, mg)	LE (%)	BSA (Green,mg)	LE (%)	Zeta potential (mV)
High PSS	1	0.2	0.05		0.25	98.5	0.25	93.4	5.0
Low PSS	1	0.2	0.01		0.25	74.2	0.25	64.3	12.1
High HA	1	0.2		0.05	0.25	97.0	0.25	97.3	3.0
Low HA	1	0.2		0.01	0.25	70.2	0.25	54.6	10.5

The optimized ratio of TMC : CSP was determined at 10:2.5 if CSP is the only protein encapsulated in the core of the nanoparticles. The CSP has found relatively more negative charge in PBS solution than BSA protein which caused the decreased NP size with lower zeta potential compared with LbL NP-BSA formulation. However, for two- and three-protein loading, CSP was loaded in the outer layer of the formulation. The average size of TMC NPs loaded with CSP was much smaller than AMA-1 due to the protein surface charge and sizes. As a result, we slightly modified the ratio of TMC to AMA-1 to 10:1.5 to achieve highly stable in PBS and uniform size distribution of NPs when we loaded both MSP-1 and CSP LbL in one formulation. As shown in the **Table 3**, loaded with multiple proteins, the average size was increased to 305.5 nm for two layers of protein loading and 339.1 nm for three-layer protein loading, however, which were each found to be stable at an acceptable size range for intramuscular injection. These three formulations that were used in the animal studies.

**Table 3:** Parameters for the formulations for the efficacy studies.

Formulations(Main components)	Ratio of each component (weight ratio)	DLS (nm)	Zeta potential
LbL NP	10	129.1± 45.6	28.1 ± 5.8
LbL NP-CSP	10:2.5	236.6 ± 97.6	14.2 ± 9.3
LbL NP-AMA-CSP	10:1.5:1.5	305.5 ± 92.6	13.2 ± 5.6
LbL NP-AMA-MSP-CSP	10:1.5:1.5:1.5	339.1 ± 121.4	11.2 ± 4.9

### 3.3 Evaluate the Antigenicity and Integrity of Antigen Loaded Chitosan NPs Complexes

**Figure 5:**
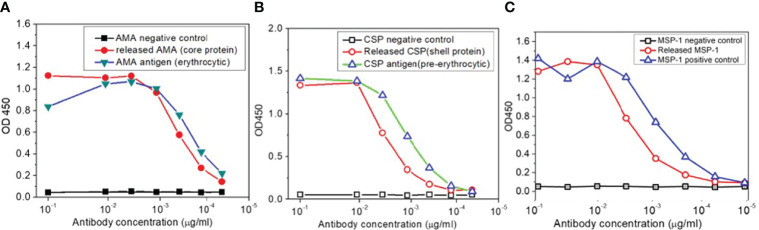
**(A)** ELISA antigenicity test for comparison of released AMA, **(B)** CSP and **(C)** MSP-1 antigens from TMC nanoparticles with corresponding malaria antigens.

LbL NP malaria antigens were prepared as shown in the Method section. To confirm that the antigenicity of each antigen on LbL NPs will not be altered following entrapment or loading on the chitosan surface. An enzyme-linked immunosorbent assay (ELISA) was used to evaluate the effect of the preparation process on released protein binding, and a nano-enhanced Surface Plasmon Resonance Image (nanoSPRi) method was used to monitor binding kinetics of release antigens. After each step of antigen loading on trimethyl chitosan nanoparticles, the antigens were released and collected for evaluation. From ELISA results shown in **Figures 5** **A–C**), we observed that released AMA-1, CSP, and MSP-1 showed similar ELISA profiles as control antigens without any processing to the antibody responses.

**Figure 6:**
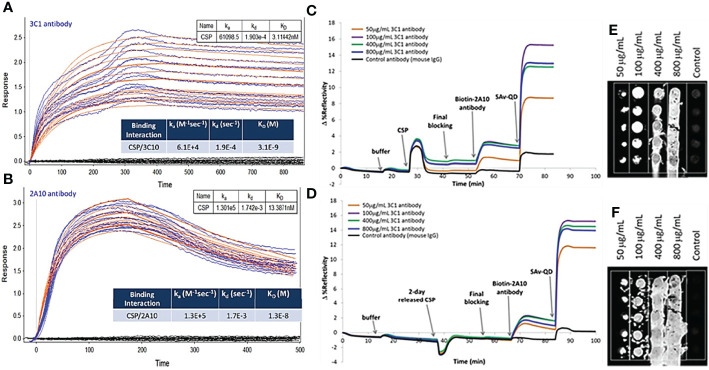
Sensorgram fits and corresponding images of **(A, B)** CSP/3C1 and CSP/2A10 malaria antigen/antibody pairs using a 1:1 biomolecular interaction model to obtain kinetic parameters ka, kd and KD as shown in the insert Table. Anti-CSP antibody (3C1 or 2A10) was spotted as capture antibody (CAb) in 4 different concentrations. CSP protein (10 µg/mL) was then injected in the flow cell and allowed to interact with the immobilized CAb on the chip surface to determine association and dissociation of protein. **(C, E)** Sensorgrams for the detection of CSP and **(D, F)** 2-day released CSP from chitosan nanoparticles. Anti-CSP 3C1 as a capture antibody while anti-CSP 2A10 as detection antibody.

We next evaluated malaria antigens using nanoSPRi to confirm that the antigen has similar binding kinetics and affinity to the correlated antibodies before and after loading and released from the NPs. Binding strength/affinity of malaria protein to their respective ligand (i.e., antibody) is an important factor when determining the efficacy of the developed vaccine. It is related to a kinetic parameter KD, the ratio of dissociation to association constants (kd/ka) of the antigen/antibody interaction. The lower the KD value, the higher the affinity of the antibody to its antigen. For this test, we focused on PfCSP as the representative target. The binding kinetics of malaria antigen CSP with its antibody pair was determined using nanoSPRi platform. Here, we chose a specific monoclonal antibody (3C1) that recognize the central repeat and C-terminal regions of PfCSP and a gold standard monoclonal antibody 2A10 (7). As shown in **Figures 6A, B**, the sensorgram fit for CSP/3C1 (antibody) binding interaction was more pronounced when compared to that of CSP/2A10 (antibody). The calculated KD values for CSP/3C1 and CSP/2A10 antigen/antibody pairs were 3.1×10^-9^ M and 1.3×10^-8^ M, respectively. This indicates that the binding strength of CSP/3C1 pair was stronger than that of a CSP/2A10 set. These results demonstrated that both antibodies could be used to obtain a sandwich construct for malaria detection. We next evaluated the binding interactions of the released CSP (rCSP) from chitosan. Results are shown in **Figures 6C–F** of the sensorgrams for detection of CSP. No significant increase of antigen signal (delta value of 15) before and after the CSP release was observed. These results indicate that the released CSP maintained its binding function and was very similar to the original CSP.

### 3.4 Perform Animal Safety Studies

**Figure 7:**
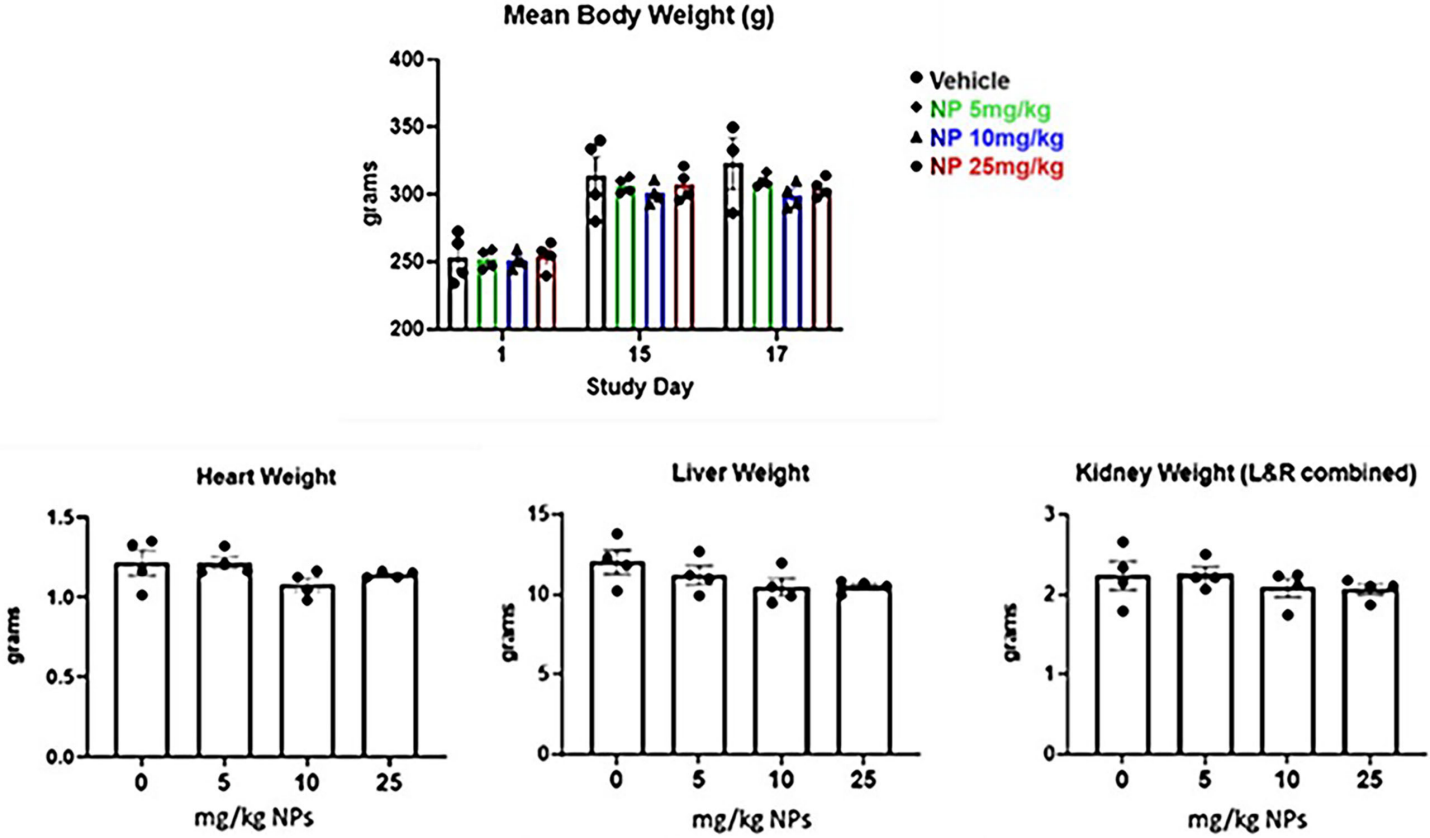
Mean body weight and organ weight changes over time after IM injection of two doses of LbL NP at concentration of 0-25mg/kg in Sprague‐Dawley rats.

There were no significant concerns, or any nanoparticle related clinical observations found during the safety studies using Sprague-Dawley rats after administration of 0-25 mg/kg dose of nanoparticles. Only we found several animals exhibited bruising or scabbing of the tail as a result of tail vein blood collections. There were also no statistically significant differences in mean body weights between groups on Day 1, 15, and 17 (**Figure 7**). In addition, there were no differences in heart, liver, or kidney weight between groups. We also evaluated the hematology, clinical chemistry, and histopathology parameters after administration of different doses of nanoparticles. While not statistically significant, white blood cell counts appeared elevated in nanoparticle treated animals compared with vehicle controls. Albumin decreased in a dose-dependent manner while globulin increased in treated animals. These alterations likely resulted from an inflammatory process. Na/K ratios increased in a dose-dependent manner, possibly due to transcellular shifts or increased renal excretion following induction of initial hyperkalemia upon skeletal muscle damage. Remaining parameters (total proteins, hemoglobin, MCV, MCH, platelets, MPV, neutrophils, lymphocytes, monocytes, etc.) were comparable across groups in this short-term study. While vehicle control skeletal muscle was normal, nanoparticle-treated animals had similarly affected hind limb lesions at the site of injection. The muscle bundles were dissected by edema and inflammation. Heart, kidney, and liver were normal in all study animals. This result provides safety dose range for immunogenicity and efficacy studies at below 5 mg/kg. We used 2.2 mg/kg dose for nanoparticle delivery antigens which is far below the safety dose ranges in the immunogenicity and efficacy study.

### 3.5 Mouse Immunogenicity Studies of Chitosan Loaded Multiple Stage Antigen Releases

We formulated vaccine candidates by loading pre-erythrocytic protective antigen CSP and blood stage protective antigen AMA-1, MSP-1 in the LbL NP (**Table 1** and Section 3.2 for details). After dosing, mouse sera were collected for serology analysis of the antibody titers using ELISA of CSP, MSP1 and AMA1 for each formulation and the numbers of IFN-γ-secreting T cells in spleens of mice immunized with antigens by intramuscular injection were measured by IFN- γ enzyme linked immunospot (ELISPOT) assay.

**Figure 8:**
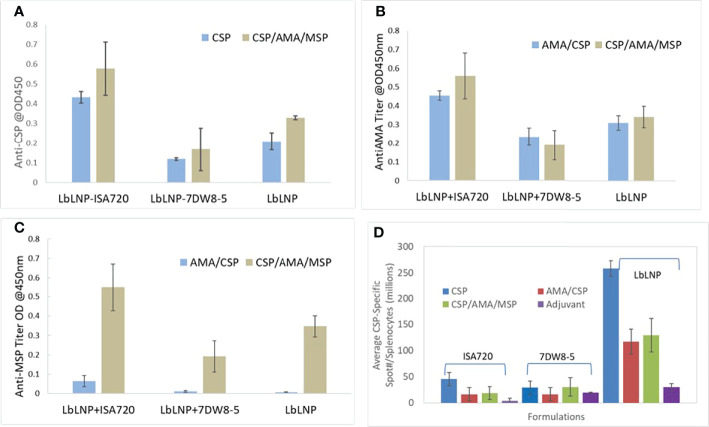
ELISA of anti-CSP **(A)**, anti-AMA-1 **(B)** and anti-MSP-1 **(C)** induced by 2 doses of immunization of different vaccine candidates incorporated with and without adjuvant ISA 720 and 7DW8-5. Each group has four mice, and the data were averaged from these 4 mouse sera at 20,000 dilutions. **(D)** PfCSP of P. falciparum -specific T cell responses (IFN-γ ELISPOT) induced by immunization with LbL NP formulations and compared with two other adjuvants ISA 720 and 7DW8-5 using in the vaccine formulations for BALB/c mice study by 2-dose intramuscular injection.

From the cellular response and ELISA results shown in **Figures 8** **A–C**), it was demonstrated that LbL NP possessed an adjuvant effect. Especially in the group with adjuvant ISA 720 (see **Table 1** for details), it produced the highest humoral responses after administrated two doses of vaccine candidates in sera samples (20,000 dilutions). Also, we found out that in the 2-dose administration, this should be enough dosing to induce sufficient response compared to 3-doses for all the antigens. In the LbL NP group, we found the three-antigen loaded formulation with 2-doses demonstrated the highest humoral responses in sera samples but not for other combination adjuvant groups. However, injection of unloaded LbL NP alone as the negative control also resulted in a very low responses (<0.05) of antibody against PfCSP. Results from the ELISPOT CD4 T-cell response study demonstrated that LbL NP group formulations showed greater responses in CSP-specific CD4 T-cells than the other two adjuvant groups (ISA72 and 7DW8-5, **Figure 8**). In the 3-dose results, these responses continued to increase for the NP-CSP formulations (data not shown here). The NP vaccine candidate group alone induced the highest PfCSP specific T-cell response. We inferred that chitosan nanoparticles have a sugar like structure which is similar to the PfCSP sugar structure and is likely to induce higher cellular responses. This statement of potential adjuvant effect will be further investigated in future studies. The LbL NP vaccine candidate is such a potent immunogen that it may induce antibodies that have reactivity to CSP, AMA-1 and MSP. So far, we have concluded that the LbL NP is an extremely potent vaccine vector. 2-doses of immunization with a longer interval between them, likely 4 weeks, has potentially shown to induce the highest humoral response against CSP. We worked on this dosing plan in the efficacy studies.

### 3.6 Perform Protective Efficacy Studies by Parasite Challenge in Mouse Model

We next investigated the efficacy of LbL NP-antigen vaccine candidates to protect against sporozoite challenge in a mouse model, as analyzed by the presence or absence of parasitemia in thin blood smears. Intramuscular injection was used as the administration route and Malaria proteins were formulated using LbL NP and other two adjuvants for comparison. For the efficacy studies, both immunized and naïve mice were challenged intravenously with a recombinant rodent malaria strain, *Plasmodium yoelii* parasite that bears a full-length PfCSP, called PfCSP/Py sporozoite (Spz) (6), kindly provided by Sanaria Inc.

First, we injected 3 groups (N=4 per group) of female BALB/c mice intravenously with 375, 750, and 1500 Spz of the PfCSP/Py parasites. The infectivity of PfCSP/Py Spz was determined by the presence or absence of parasites (parasitemia) in the blood of the challenged mice. All the mice that received 1500, as well as 750 spz, developed parasitemia by Day 7 post challenge. However, three out of four mice that received 375 spz failed to develop parasitemia. Therefore, we challenged vaccinated mice with 1000 PfCSP/Py Spz in the experiments to investigate efficacy.

Single (CSP) and three-protein (AMA-1/MSP-1/CSP) LbL NP formulations were prepared by ionic gelation using the developed method (Method in section 2). The optimized ratio of TMC : CSP was determined at 10:2.5 if CSP is the only protein encapsulated in the core of the nanoparticles. For three-protein loading, CSP was loaded in the outer layer of the formulation. For three protein formulations, the ratio of TMC to CSP, AMA-1 and MSP-1 was 10:1.5:1.5:1.5 which were described in Section 3.2 and **Table 3**. As can be seen in **Table 4** efficacy results, all naïve mice were successfully infected with transgenic malaria parasites. Five out of six mice immunized with adjuvanted CSP (Groups 2, 5, 8) were protected (100-83.3% protection) compared to the naïve mouse group, indicating that the single antigen CSP vaccine displayed good efficacy. Immunization with CSP/AMA1/MSP1 with 7DW8-5 (Group 6) and NP-CSP/AMA1/MSP1 (Group 9) also induced a moderate protection (66.7%) compared to the naïve mouse group, resulting in protection for four out of six mice. When we compared these vaccinated groups with internal control groups (adjuvant alone Groups 7 and 10), CSP + 7DW8-5 (Group 5), and NP-CSP (Group 8) still demonstrate statistically significant efficacy (p<0.05, Fisher’s test). However, immunization with CSP/AMA1/MSP1 with ISA720 (Group 3) was able to protect only two out of six mice (33.3%), which was identical to that seen in mice immunized with ISA720 alone (Group 4). The reason why CSP alone seems more potent is likely because when more than one protein is combined, the presentation of a single antigen could be slightly diminished due to competition at the level of antigen-presentation, as multiple proteins will compete for MHC class I and class II-mediated presentation.

**Table 4:** Protection of mice immunized with vaccine candidates against transgenic PfCSP/Py sporozoites administrated intravenously.

Vaccine formulations	Protected/Challenged	p Value
Group 1: Naive	0/6	
Group 2: CSP+ISA720	6/6	0.061^a^
Group 3: CSP/AMA1/MSP1+ISA720	2/6	1^a^; 0.24^b^
Group 4: ISA720 only	2/6	
Group 5: CSP+7DW8-5	5/6	0.008^a^*; 0.015^b^*
Group 6: CSP/AMA1/MSP1+7DW8-5	4/6	0.24^a^; 0.061^b^
Group 7: 7DW8-5 only	1/6	
Group 8: CSP+LbL NPs	5/6	0. 008^a^*; 0.015^b^*
Group 9: CSP/AMA1/MSP1+LbL NPs	4/6	0.24^a^; 0.061^b^
Group 10: LbL NPs only	1/6	

a The p value between vaccine candidate group to each adjuvant group.

b The p value between vaccine candidate group to naïve group.

*p<0.05 significant.

## 4 Discussion

It is important that the delivery vehicle used for antigen delivery is highly stable and uniformly dispersed in the human blood system. The limited solubility of chitosan and chitosan-based materials therefore hinders its use and application for a wide range of biological environments. The reductive methylation of chitosan for obtaining N, N, N-trimethyl chitosan (TMC) is a good strategy for overcoming such limitations because TMC can be soluble in distilled water, in PBS solution, and in alkaline or acidic aqueous solutions. The solubility of TMC across the range of pH is due to the shifting in charge density originated by methylation of primary amino groups on chitosan. Also, the methylation of chitosan results in a high positive charge on the surface of TMC which is beneficial for loading of negatively charged biological molecules. FTIR and NMR analysis of our synthesis indicates that the TMC was successfully prepared and trimethylation was successful.

The ionic gelation method is considered the most suitable method for protein loading on TMC NPs. The presence of cross linker TPP and surface coating chemistries of PSS or HA have been evaluated to select the optimized composition for development of malaria vaccine candidates. The use of TPP as a crosslinker was found necessary for successful protein encapsulation within the core of the nanoparticle. PSS as a surface protection coating was found to moderately decrease the loading amount of core and second layer proteins. However, this was determined acceptable since it is used as a protective layer to prevent outer layer proteins from immediate release and degradation. PSS has a very strong affinity to TMC as compared to HA during the formation of NPs. As a result, if we apply PSS as the protective layer, the amount of PSS needs to be limited to less than 0.05 mg per mg of TMC to avoid large precipitations. Release testing also confirmed that undesired burst release was lower if we decreased the protect layer PSS amount. HA performed similar function as PSS, but it provided more benefits that it reduced precipitation of nanoparticle when we used the same amount of coating as PSS. And it also helped in prolonged the release of loaded protein compared with using PSS as the protected layer. HA could be the most beneficial for the formation of LbL NP vaccine candidate. Combined, we concluded that the protective layer is necessary and required for achieving long-term release profiles.

Three multiple stage malaria life cycle antigens were successfully encapsulated and loaded on TMC nanoparticles. These antigens are pre-erythrocytic stage antigens (i) circumsporozoite protein (CSP; the major antigen on the sporozoite surface and its fragments have been included in the most clinically advanced malaria vaccine RTS, S) (12). However, RTS, S does not include the N-terminal region of CSP. Adoptive transfer of a monoclonal antibody specific for the N-terminus of the P. falciparum CSP, strongly inhibits the infection of rodent malaria sporozoites expressing the N-terminus of P. falciparum CSP (13). The erythrocytic stage antigens (ii) apical membrane antigen 1 (AMA1; involved in merozoite invasion of red blood cells and essential to the proliferation and survival of the malarial parasite, and its antibodies have shown to be protective (14), and (iii) merozoite surface protein 1 (MSP1; highly immunogenic in humans and numerous studies suggest it is an effective target for a protective immune response (15). We worked with GenScript for synthesis of the plasmid to produce full size of CSP protein. As widely known, a wide variety of factors regulate and influence gene expression levels, and GenScript used OptimumGene™ algorithm to consider as many of these factors as possible, producing the single gene that can reach the highest possible level of expression. In this case, the native gene employs tandem rare codons that can reduce the efficiency of translation or even disengage the translational machinery. They increased the codon usage bias in *E. coli* by upgrading the Codon Adaptation Index (CAI) to 0.74. CAI of 1.0 is considered to be perfect in the desired expression organism.

ELISA results demonstrated that both free and entrapped protein after release from NPs possessed similar responses to their antibodies. Binding strength/affinity of released proteins to their respective receptors is an important factor when determining the efficacy of the developed vaccine. Binding, specificity, affinity, kinetics, and active binding concentration were determined from the shape of produced surface plasmon resonance imaging sensorgrams. These observations demonstrated that released PfCSP maintained binding properties to corresponding antibodies 2A10 and 3C1 following loading and release in the LbL NPs.

Many candidate vaccines evaluated to date fail to achieve protection against certain human pathogens, such as malaria, and this is primarily due to their poor cellular immunogenicity (16). As a result, it is important that newly developed adjuvant LbL NP may add value when it is used as a stand-alone manner or in combination with existing adjuvants such as ISA 720 (9) and 7DW8-5 (16). Here, we found *in vivo* immunogenicity tests using LbL NPs as antigen/protein delivery vehicles demonstrated immunoadjuvant properties. The LbL NP formulation groups showed the greatest PfCSP specific T-cell responses in mice and also strong titers of humoral responses. Specific IgG was detected in all mice receiving vaccine formulation with sera dilutions between 4,000 and 20,000 after 2 doses. Finally, we challenged with *P. yoelli* parasites that express only PfCSP, and therefore, we saw the protective immune response targeted against PfCSP only. When we determined the level of protection by the presence or absence of parasitemia in thin blood smears, we found that 5 of 6 mice were protected against malaria challenge after boost of LbL NP delivery of full length of CSP as the vaccine candidate. Thus, we systematically demonstrated that intramuscular injection of LbL NP leads to a more potent adjuvant effect than commercial ISA720 in the efficacy studies. However, if we establish PfCSP/PfAMA-1/PfMSP-1 triple transgenic parasites and challenge them, the LbL NP expressing the three proteins may exert a better efficacy compared to a single protein-expressing LbL NP vaccine. Although the protective immunity induced by PfCSP (one antigen) may be weaker, a combined protective immunity induced by all 3 proteins may be more potent due to additive or synergistic effect. Also, it is rare to see protection lasting for more than 4 weeks after a booster dose. To the best of our knowledge, there have been no other malaria vaccines found that can sustain sterile protection for more than 2 weeks.

Also, we observed that LbL NPs are potentially a good adjuvant candidate for vaccine delivery in order to obtain long-lasting protection. The LbL NP found may elicit innate immune response that was potent to mediating non-specific anti-malarial effect. In the safety studies, we found LbL NP at a dose of less than 5 mg/ml were also determined biocompatible and safe in male Sprague-Dawley rats. While these studies suggest a protective response using LBL NP as the delivery vector, additional studies are necessary to fully understand the potential of the nanoparticle approach due to the smaller number of mice per group in this study.

## Data Availability Statement

The original contributions presented in the study are included in the article/**Supplementary Material**. Further inquiries can be directed to the corresponding author.

## Ethics Statement

The animal study was reviewed and approved by the Institutional Animal Care and Use Committee (IACUC) at The Columbia University (Animal Welfare Assurance no. D16-00003) and Michigan State University (Animal Welfare Assurance no. A3955-01).

## Author Contributions

Conceptualization by YX. Methodology was done by ZZ, BB, TF. JO, HZ, XJ, CL prepared the immunogens and evaluated the antigen functionality. IK, SI, MY have made the transgenic PfCSP/Py Spz. JH, YT and MT have conducted the immunogenicity and efficacy studies in mice. YX drafted the manuscript, and CT, X-PK and MT participated in reviewing and editing the manuscript. All authors contributed to the article and approved the submitted version.

## Funding

This material is based upon work supported by the US Army Medical Research and Materiel Command under Contract No W81XWH-18-C-0073. And also it is supported by the Army/DHA SBIR Program/US Army Medical Research and Development Command (USAMRDC)/Walter Reed Army Institute of Research (WRAIR), Military Infectious Diseases Research Program (MIDRP)/JPC-2 under Contract No. W81XWH21C0057.

## Acknowledgments

We thank Dr. Matthew Bernard and Dr. Krieger-Burke Teresa at the Michigan State University *In Vivo* Facility for nanoparticle safety and tolerance studies. We thank Sanaria, Inc. for kindly provide us with cryopreserved Pf/Py Spz.

## Conflict of Interest

Authors YX, ZZ, BB, TF, JO and CT were employed by company Luna Labs USA.

The remaining authors declare that the research was conducted in the absence of any commercial or financial relationships that could be construed as a potential conflict of interest.

## Publisher’s Note

All claims expressed in this article are solely those of the authors and do not necessarily represent those of their affiliated organizations, or those of the publisher, the editors and the reviewers. Any product that may be evaluated in this article, or claim that may be made by its manufacturer, is not guaranteed or endorsed by the publisher.
