# Supplementary material for: Layer-by-Layer Delivery of Multiple Antigens Using Trimethyl Chitosan Nanoparticles as a Malaria Vaccine Candidate
Source: Front Immunol. 2022 Aug 17;13:900080. doi: 10.3389/fimmu.2022.900080 (PMC9428560; doi:10.3389/fimmu.2022.900080)
Supplement: Supplementary file 1 [file DataSheet_1.docx]

Supplemental information

S-Figure 1. Design of Luna’s nanoSPRi-based assay for malaria antigen detection. This assay is run in the flow cell of an OpenPlex SPRi instrument from HORIBA Scientific. A malaria antigen is captured by the specific capture antibody array, detected by the biotinylated antibody, and amplified using a quantum dot nano enhancer. The concentration and real-time binding kinetics of antigen to its antibody are determined from changes in reflectivity.


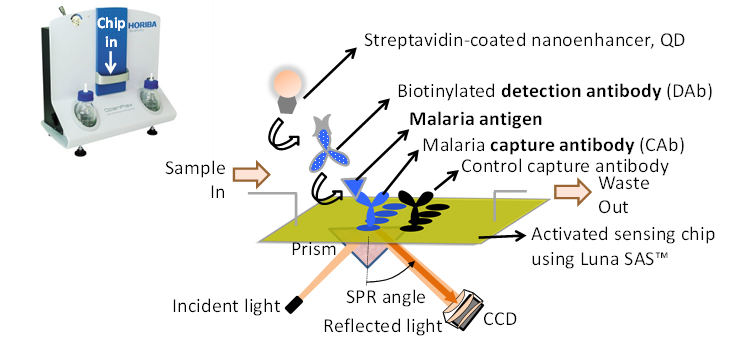


S-Figure 2. Average size of TMC-TPP single nanoparticle, self-assembly nanoparticle at different reaction time, and LbL NP-protein complex.


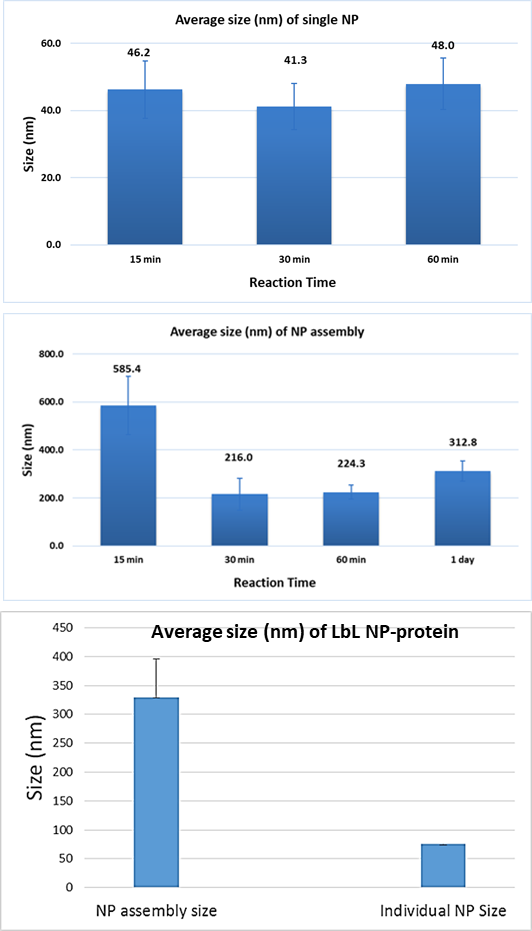


S-Table 1. Study schedule and dose ranges for IM administration of rat.

| Group | Sex | n | Animal No. | TMC/TPP-NPs (mg/kg) |
| --- | --- | --- | --- | --- |
| 1 (control) | Male | 4 | 1001-1004 | 0 (vehicle) |
| 2 (low) | Male | 4 | 2001-2004 | 5 |
| 3 (moderate) | Male | 4 | 3001-3004 | 10 |
| 4 (high) | Male | 4 | 4001-4004 | 25 |

S-Table 2. Parameters of NP (TMC-PSS) and related zeta-potential value of NPs

| **Ratio of TMC: PSS** | **Reaction time (min)** | **TMC (mg)** | **TPP (mg)** | **PSS (mg)** | **zeta-potential (mV)** |
| --- | --- | --- | --- | --- | --- |
| 1: 2 | 60 | 0.5 | 0 | 1 | -37 |
| 1: 1 | 60 | 1 | 0 | 1 | -27 |
| 1:0.91 | 60 | 1.1 | 0 | 1 | -23 |
| 1:0.83 | 60 | 1.2 | 0 | 1 | 23 |
| 1:0.77 | 60 | 1.3 | 0 | 1 | 19 |
| 1:0.71 | 60 | 1.4 | 0 | 1 | 25 |
| 1:0.67 | 60 | 1.5 | 0 | 1 | 30 |
| 1:0.5 | 60 | 2 | 0 | 1 | 35 |

S-Table 3. Parameters of NP (TMC-TPP-PSS) and related zeta-potential value of NPs

| **Ratio**  **(TMC: TPP: PSS)** | **Reaction time (min)** | **TMC**  **(mg)** | **TPP**  **(mg)** | **PSS**  **(mg)** | **zeta-potential**  **(mV)** |
| --- | --- | --- | --- | --- | --- |
| 1:0.2:0.1 | 60 | 1 | 0.2 | 0.1 | 5 |
| 1:0.2:0.25 | 60 | 1 | 0.2 | 0.25 | 4 |
| 1:0.2:0.5 | 60 | 1 | 0.2 | 0.5 | 4 |
| 1:0.2:0.75 | 60 | 1 | 0.2 | 0.75 | -23 |
